# Reversal of Muscle Atrophy by Zhimu-Huangbai Herb-Pair via Akt/mTOR/FoxO3 Signal Pathway in Streptozotocin-Induced Diabetic Mice

**DOI:** 10.1371/journal.pone.0100918

**Published:** 2014-06-26

**Authors:** Jinbao Zhang, Pengwei Zhuang, Yan Wang, Lili Song, Mixia Zhang, Zhiqiang Lu, Lu Zhang, Jing Wang, Paulos N. Alemu, Yanjun Zhang, Hongjun Wei, Hongyan Li

**Affiliations:** 1 Chinese Materia Medica College, Tianjin University of Traditional Chinese Medicine, Tianjin, China; 2 Tianjin State Key Laboratory of Modern Chinese Medicine, Tianjin University of Traditional Chinese Medicine, Tianjin, China; 3 Tianjin JF-Pharmaland Technology Development Co., Ltd., Tianjin, China; Universidad Pablo de Olavide, Centro Andaluz de Biología del Desarrollo-CSIC, Spain

## Abstract

Skeletal muscle atrophy is one of the serious complications of diabetes. Zhimu-Huangbai herb-pair (ZB) is widely used in Chinese traditional medicine formulas for treating Xiaoke (known as diabetes) and its complications. However, the effect of ZB on reversal of muscle atrophy and the underlying mechanisms remain unknown. In this research, we investigated the effect and possible mechanisms of ZB on skeletal muscle atrophy in diabetic mice. Animal model of diabetic muscle atrophy was developed by high fat diet (HFD) feeding plus streptozotocin (STZ) injection. After oral adminstration of ZB for 6 weeks, the effects of ZB on reversal of muscle atrophy and the underlying mechanisms were evaluated by biochemical, histological and western blot methods. The skeletal muscle weight, strength, and cross-sectional area of diabetic mice were significantly increased by ZB treatment. Biochemical results showed that ZB treatment reduced the serum glucose level, and elevated the serum insulin-like growth factor 1 (IGF-1) and insulin levels significantly compared with untreated diabetic group. The western blot results showed that ZB activated the mTOR signal pathway, shown as increased phosphorylations (p-) of Akt, mTOR, Raptor, S6K1 and reduced Foxo3 expression compared with the model group. ZB could reverse muscle atrophy in diabetic mice. This may be through activation of mTOR signaling pathway that promotes protein synthesis, and inactivation foxo3 protein that inhibits protein degradation. These findings suggested that ZB may be considered as a potential candidate drug in treatment of diabetic muscle atrophy.

## Introduction

Muscle atrophy is defined as a decrease in the mass of the muscle. It occurs in diabetes and other pathological conditions, including cancer, sepsis, and renal failure [1]–[3]. Normal daily activity needs adequate muscle size and strength, and muscle atrophy has a negative effect on overall quality of life. Diabetes mellitus can cause skeletal muscle damage and atrophy by the direct effects of high glucose and low insulin [4]. Muscle wasting in diabetes is ultimately the result of damage to the intracellular signaling pathways that are involved in maintaining the balance between synthesis and degradation of protein [5], [6]. Many researches have proved that IGF-1 can inhibit skeletal muscle atrophy by inreasing protein synthesis via activation Akt/mTOR pathways [7]–[9], Besides, IGF-1 can prevent skeletal muscle atrophy by inhibiting protein degradation via the Akt/FoxO pathways [7], [9]–[11].

Mammalian target of rapamycin (mTOR) is a key regulator of protein synthesis. It has been widely confirmed that signaling pathway of mTOR is both necessary and sufficient for the induction of skeletal muscle hypertrophy [12]. The Akt/mTOR pathway is closely related with muscle hypertrophy and atrophy. Activation of the Akt/mTOR pathway can oppose muscle atrophy [13]. Muscle-specific inactivation of mTOR leads to severe myopathy [14]. mTOR contains two distinct multiprotein complexes known as mTOR complex 1 (mTORC1) and mTOR complex 2 (mTORC2) [14], [15]. The activation of mTORC1 promotes protein synthesis, lipogenesis, energy metabolism, and inhibits autophagy and lysosome biogenesis. Alternatively, the mTORC2 activated by growth factors regulates cytoskeletal organization and cell survival/metabolism [16]. The mTORC1, which includes Raptor, signals to S6 kinase 1 (S6K1) and 4EBP1, is rapamycin-sensitive. mTORC2, which includes Rictor, is rapamycin-insensitive [17]. Many researches have elucidated that the mTORC1 is an important regulatory in controlling muscle protein synthesis [15], [18]. In addition, Bentzinger’s research showed that the mTORC1 component raptor is critical for muscle function and prolonged survival. Moreover, they found that skeletal muscle-specific ablation of raptor causes metabolic changes and results in muscle dystrophy [19]. S6K1 is essential for the control of muscle cytoplasmic volume by Akt/mTOR. Deletion of S6K1 will reduce myoblast size to the same extent as that observed with mTOR inhibition by rapamycin [20].

Muscle atrophy occurs when the degradation rate is higher than the synthesis rate [21], [22]. Protein synthesis mediated by mTOR is activated by Akt, whereas protein degradation mediated by the forkhead box O (FoxO) transcription factors is suppressed [23]. The transcription factors of the FoxO family are already recognized as a major regulator of the muscle atrophy program. FoxO3 is activated during muscle atrophy, and its overexpression is able to reduce muscle mass, since it activates the expression of ubiquitin ligase Atrogin-1 [22], [24]. Two muscle-specific ubiquitin ligases, Atrogin1/MAFbx and MuRF1, are induced during atrophy and are responsible for the loss of muscle mass [22], [25], [26]. Thus, FoxO play a critical role in the development of muscle atrophy, and inhibition of FoxO factors is an attractive approach to resist muscle wasting [2], [22].

For thousands of years, Traditional Chinese Medicine has played an indispensable role in the prevention and treatment of diseases in China. Moreover, many traditional medicinal herbs have been used to treat diabetes and abundant experience has been accumulated [27], [28]. Herb pairs are the most fundamental and the simplest form of multi-herb formula. Zhimu-Huangbai herb-pair (ZB) is a famous formula originated from LiaoBenZiShenWan recorded in Lanshimicang written by Li Dongyuan [29]. ZB is composed of two herbal medicines, Rhizoma Anemarrhena and Cortex Phellodendri. The main chemical constituents of ZB have been well investigated in treatment of diabetes or diabetic complications [30]–[35]. However, the effect of ZB on diabetic myopathy is unclear. Hence, we investigated the effects of ZB on muscle atrophy and the underlying molecular mechanisms in STZ-induced diabetic mice.

## Methods

### Preparation of extracts

Rhizoma Anemarrhena and Cortex Phellodendri were collected from the regions of Anguo City in Hebei Province, China. The field studies did not involve endangered or protected species, no specific permissions were required for these locations/activities. And the researches were finished in Tianjin University of Traditional Chinese Medicine, No. 312 Anshanxi Road, Nankai District, Tianjin 300193, China. The herbs were authenticated as the dried rhizome of *Anemarrhena asphodeloides* Bge and dried rhizome of *Phellodendron amurense* by senior botanist Dr. Tianxiang Li. Rhizoma Anemarrhena (100 g) and Cortex Phellodendri (100 g) were reflux extracted with 50% ethanol (2000 ml) three times for 1 h each time respectively. The filtered solutions were combined and concentrated by rotary evaporation to 100 ml (1 ml equivalent to 1 g of the crude drug). And the main components of the extracts were detected by UPLC/Q-TOF-MS system (Fig. S1).

### Animals

Twelve-week-old male C57BL/6J mice were purchased from Vital River Laboratory Animal Technology Co., Ltd. (Beijing, China). Animals were kept in an environmentally controlled breeding room (temperature: 22±2°C, humidity: 60±5%, 12 h dark/light cycle). Water and food were provided ad libitum. The study was carried out in strict accordance with the recommendations in the Guide for the Care and Use of Laboratory Animals of Institutional Animal Care and Use Committee of Tianjin University of Traditional Chinese Medicine. The protocol was approved by the Animal Ethics Committee of Tianjin University of Traditional Chinese Medicine (No.TCM-2012-010-E01), China.

### Generation of diabetic model and treatment with drug

The method and procedures are almost the same as previous reports [36], [37]. The mice were placed on HFD D12492 (Research Diets, New Brunswick, NJ), in which 60% of kilocalories is from fat. After 3 weeks of HFD feeding, the mice were intraperitoneal injected once with low-dose STZ (100 mg kg^−1^ body weight in 0.1 M sodium citrate buffer, pH 4.5; Sigma-Aldrich, St Louis, MO, USA) to induce partial insulin deficiency. The normal diet-fed mice were injected once with vehicle citrate buffer. Three weeks after STZ injection, animals with similar degrees of hyperglycemia and body weight were randomly divided to model and treatment groups termed as M and ZB group respectively. The normal diet-fed mice were used as nondiabetic control termed as C group.

ZB was administered to the mice by gavage at a dose of 0.1 ml/10 g (2.6 g crude drug/kg body weight) per day for six weeks, whereas the C and M groups were received normal saline. In the duration of treatment, the M and ZB groups were still fed with the HFD. Fasting glucose levels and body weight were monitored weekly.

### Oral glucose tolerance tests

Oral glucose load was administered at 2 g kg^−1^ of body weight after overnight fasting. Glucose levels were measured from tail bleeds at the indicated time points after glucose administration. And the glucose tolerance was evaluated by calculation of the area under the curves (AUC).

### Muscle function testing

Skeletal functional performances were assessed by using rotarod and grip strength measurement. For the rotarod test [38]–[40], the mice were acclimated to the rotarod apparatus for two consecutive days prior to data collection. During acclimation the mice were placed on the rotarod twice a day for two minutes, at a constant speed of 20 rpm. If the mice fell off the cylinder before the two minutes were up, they were placed back on the cylinder. The mice were then tested three consecutive trials per day for 3 days. The rotarod was accelerated from 5 to 40 rpm over 5 min with a maximum score of 300 sec. The latency to fall was recorded and the average of three trials per mouse was calculated and analyzed. For the Grip Strength test [41]–[43], a grip strength meter (GSM) was used to measure forelimb and hindlimb grip strength. The mice were acclimated to the GSM for five minutes one day prior to data collection. Mice were allowed to grasp the grip with all limbs. The maximum amount of force exerted was recorded. This was repeated three times with a 30 sec interval between trials and the average score (grams) was calculated.

### Muscle weight and protein content

After 6 weeks of administration, mice were anesthetized by intraperitoneal injection with 10% Chloral Hydrate (350 mg/kg body) and the gastrocnemius, quadriceps muscles were quickly harvested from hind limb, and fresh muscle weights were recorded. The muscles were homogenized and the protein contents were determined with a Bradford protein assay kit (Bio-Rad) according to the manufacturer’s instructions.

### Analysis of myofiber cross-sectional area

After the end of the experiment, hind limb muscles (gastrocnemius, quadriceps) of mice were collected, fixed in 4% paraformaldehyde in 0.1 M phosphate buffer (pH 7.4). To assess muscle fiber cross-sectional area, transverse paraffinized muscle sections (6 µm) were stained with hematoxylin and eosin (H&E). Stained sections were visualized under Olympus IX 70 microscope and pictures were captured using Olympus MagnaFire digital camera and software (Olympus America, Melville, New York). Fiber cross-sectional area was measured for approximately 100 adjacent muscle fibers in each section for each mouse using Image Pro 6.0 software (Media Cybernetics, Silver Spring, MD) [44], [45].

### Serum lipid levels, insulin, IGF-1

Mice were fasted for 12 hours and collected blood from the canthus, centrifuged for 15 minutes at 3000 rpm, the serum was separated. The total cholesterol (TC), high-density lipoprotein cholesterol (HDL), low-density lipoprotein cholesterol (LDL), and triglyceride (TG) levels were measured by semi-automatic biochemical analyzer (Microlab 300, Vital Scientific Inc.). The levels of insulin, IGF-1 were analysed with enzyme-linked immunosorbent assay (ELISA). All the steps are operated strictly in accordance with the instructions.

### Western blot analysis

The method and procedures are conducted as previously reported [46], [47]. Quadriceps muscles were lysed with RIPA lysis buffer. The protein concentrations of the lysates were determined with a Bradford protein assay kit. An equal amount of protein (40 ug) was fractionated by SDS-polyacrylamide gel electrophoresis (PAGE) and transferred onto polyvinylidene difluoride membranes. Membranes were blocked for 1 h in Tween 20 Tris-base sodium (TBST) containing 5% milk followed by incubation with the appropriate primary antibody (rabbit anti-phospho-Akt Ser473, rabbit anti-Akt, rabbit anti-phospho-mTOR Ser2448, rabbit anti-mTOR, rabbit anti-phospho-rictor Thr1135, rabbit anti-rictor, rabbit anti-phospho-raptor Ser792, rabbit anti-raptor, rabbit anti-phospho-p70S6K1 Thr389, rabbit anti-p70S6K1, rabbit anti-FoxO3, diluted 1∶1000 with 5% BSA in TBST, Cell Signaling Technology, Inc.) overnight at 4°C. After 5 times washing in TBST, membranes were incubated with goat anti-rabbit horse radish peroxidase-conjugated secondary antibodies (1∶5000) for 1 h at room temperature. Protein signals were detected with ECL Western blotting detection reagents (Millipore). Images were scanned, and band intensities were quantified by densitometry (Bioquant Image Analysis, Nashville, TN). All the protein expression data were normalized by β-actin. In addition, the phospho and total kinase determinations were performed in separate gels.

### Statistical Analysis

All data were analysed using SPSS version 16 software (SPSS Inc.) and expressed as means±SD. Statistical comparison between different treatments was done by one-way ANOVA. Differences were considered statistically significant for *P<*0.05.

## Results

### Generation of a mouse model of type 2 diabetes

Previous studies [36], [37] have shown that the combination of HFD and STZ treatment can lead to disorders in glucose and lipid metabolism accompanied by impaired insulin secretion and insulin resistance. In the present study, C57BL/6J mice were fed with HFD for three weeks and then injected with a single low dose of STZ followed by continued HFD feeding for an additional three weeks. As shown in Table 1, the HFD/STZ mouse model manifested hyperglycemia and hyperlipidemia associated with insulin resistance and impaired insulin secretion, as described in previous reports. Therefore, we used this model as type 2 diabetes to study the effects of ZB on muscle atrophy and the underlying molecular mechanisms.

**Table 1:** Characteristics of the mouse model of type 2 diabetes.

	Glucose(mmol/l)	Insulin(pmol/l)	Total cholesterol(mmol/l)	Triacylglycerol(mmol/l)	Body weight(g)
Control	4.17±0.93	101.04±22.65	1.56±0.23	0.74±0.11	23.56±1.21
HFD/STZ	17.43±2.12***	84.68±21.83*	3.21±0.28**	1.41±0.33**	25.67±1.37*

Data are means±SE. C57BL/6J mice were injected with vehicle and fed SD (control) or injected with low-dose STZ and fed with HFD (STZ/HFD). Before treatment with ZB, the blood glucose, insulin, triglycerides, total cholesterol, and body weight were measured under fasting condition. * p<0.05, ** p<0.01 vs control mice.

### ZB increased body weight, reduced glucose levels and improved glucose tolerance

**Figure 1:**
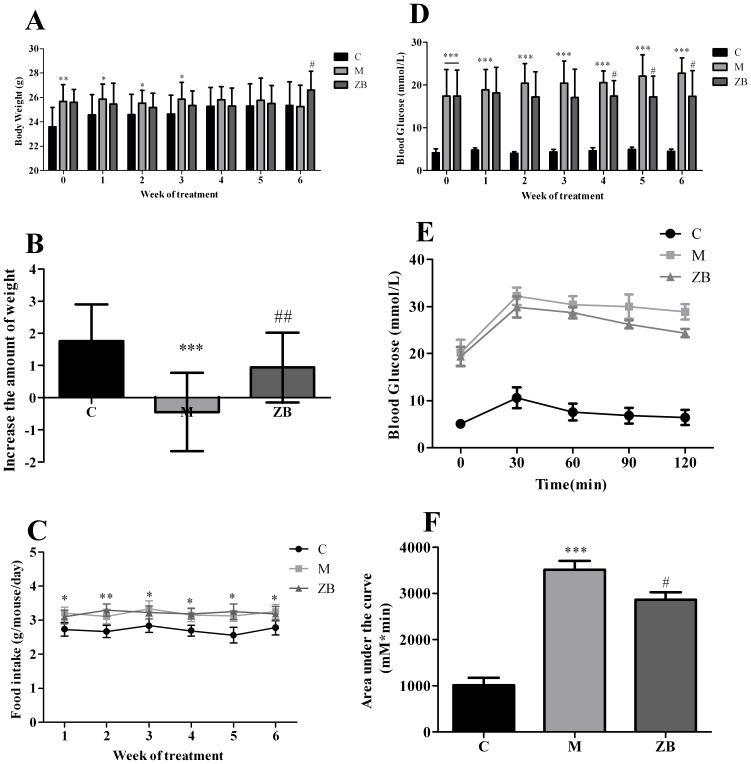
Effect of ZB on body weight, blood glucose and oral glucose tolerance of diabetic mice. A: body weight of the mice during treatment in each group. B: body weight gain of the mice during treatment in each group. C: food intake of the mice during treatment in each group. D: fasting glucose levels of the mice during treatment in each group. E: glucose tolerance test results for each group. F: area under the curve for each group. Data are expressed as the means±SD. *P<0.05, **P<0.01, ***P<0.001 vs. nondiabetic control. ^#^ *P*<0.05, ^##^ *P*<0.01 vs. Model group (*n = *15 in each group).

The ZB extract was administered to diabetic mice, the changes of body weight and fasting glucose levels were monitored weekly. Fig. 1A shows the body weights of the mice. The body weight of diabetic mice was gradually reduced, whereas the normal group steadily increased over the six weeks. However, treatment with ZB improved the disadvantaged compared with model group (*P*<0.01, Fig. 1B). The amount of food intake of the diabetic mice was significantly greater than the nondiabetic control, there was no statistical difference between model group and ZB-treated animals (Fig. 1C). Blood glucose levels in diabetic mice remained extremely high throughout the experiment (*P*<0.05, Fig. 1D). In contrast, after the fourth week, blood glucose levels of ZB-treated diabetic mice were gradually descending down until the sixth week. Oral glucose tolerance test was performed after 6 weeks of the treatment. Diabetic mice showed impaired glucose tolerance compared with C group. The ZB group displayed a significant improvement in glucose clearance, and the area under the glucose curve were reduced almost 20% compared with M group (*p*<0.01, Fig. 1E, F).

### ZB increased muscle strength and coordination

**Figure 2:**
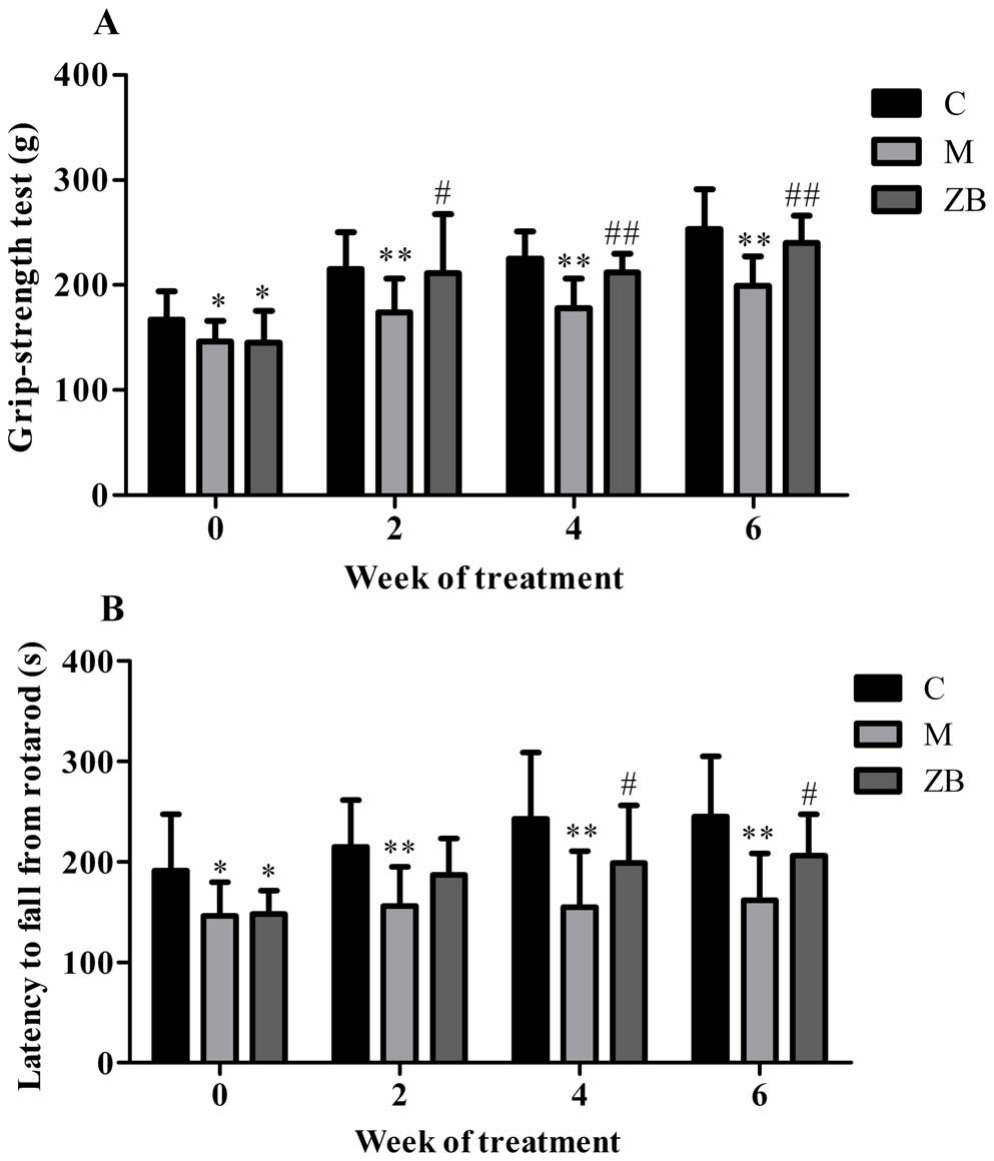
ZB increased muscle strength and coordination of diabetic mice. A: grip strength of the mice during treatment in each group. B: time to fall from an accelerating rotarod of the mice during treatment in each group. Data are expressed as the means±SD. *P<0.05, **P<0.01 vs. nondiabetic control. ^#^ *P*<0.05, ^##^ *P*<0.01 vs. Model group.

To explore the effect of ZB on muscle strength and coordination in diabetic mice, we performed grip strength and rotarod tests every 2 weeks (Fig. 2A, 2B). The model mice exhibited significant muscle weakness and tended to fall off the rotarod cylinders sooner than the control mice. ZB could increase grip strength (*P*<0.01) and prolong the time on the rotarod compared with model group (*P*<0.05).

### ZB increased muscle weight and protein content

**Figure 3:**
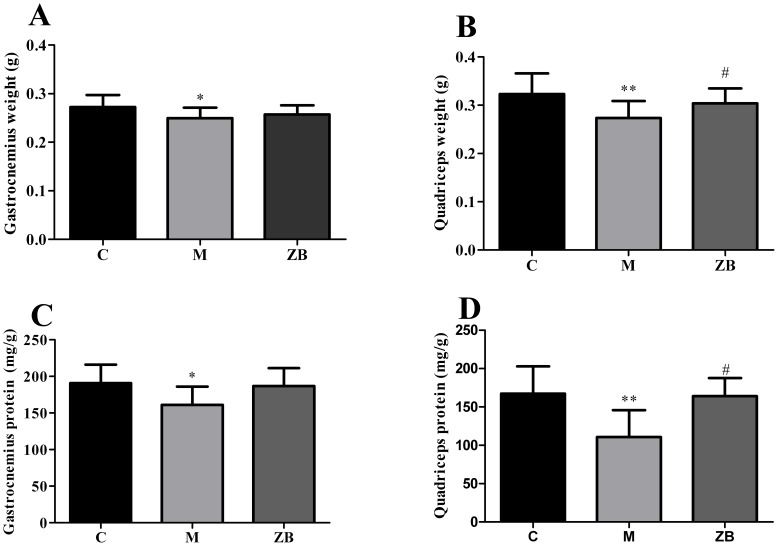
ZB increased muscle weight and protein content of diabetic mice. A: gastrocnemius weight of the mice during treatment in each group. B: quadriceps weight of the mice during treatment in each group. C: total gastrocnemius protein of the mice during treatment in each group. D: total quadriceps protein of the mice during treatment in each group. Data are expressed as the means±SD. Data are expressed as the means±SD. * *P*<0.01, ** *P*<0.01 vs. nondiabetic control. ^#^ P<0.05 vs. Model group.

We also investigated the effect of ZB on muscle weight and the total protein content. The results showed that the average gastrocnemius or quadriceps muscle weight of the model mice was significantly lower than the control mice. ZB could increase quadriceps muscle weight compared with model group (*P*<0.05) and there was no statistical significant difference in gastrocnemius muscle weight (Fig. 3A, 3B). Furthermore, the total protein content of the model mice was significantly lower than the control mice. ZB increased the total protein content of quadriceps muscle compared with model group (*P*<0.05, Fig. 3C, 3D).

### ZB increased myofiber cross-sectional area

**Figure 4:**
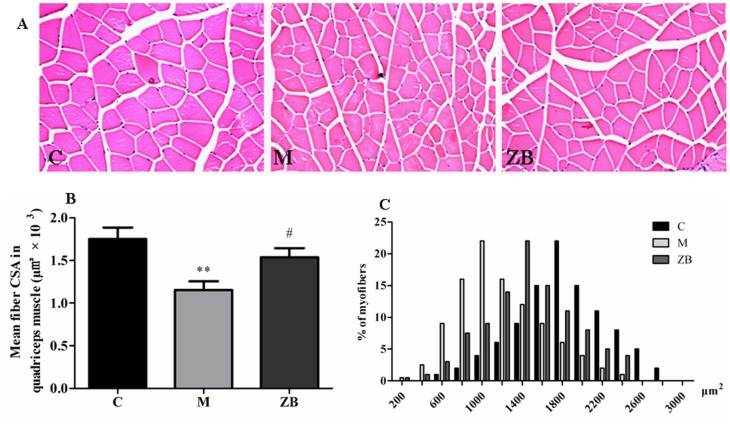
ZB increased myofiber cross-sectional area in diabetic mice. A: representative photomicrograph of H&E staining of quadriceps muscle section. B: Quantitative estimation of myofibers fiber cross-sectional area of quadriceps muscle. C: the distribution of myofiber sizes for each group. Data are expressed as the means±SD. ** *P*<0.01 vs. nondiabetic control. ^#^ *P*<0.05 vs. Model group.

To evaluate the role of ZB on the cross-sectional area of quadriceps muscles, H&E staining was performed on quadriceps muscle transverse paraffinized sections (Fig. 4A). Fiber cross-sectional areas were measured for 100 adjacent muscle fibers in each section for each mouse by Image Pro 6.0 software (Fig. 4B). The cross-sectional area of muscle fibers in model mice was significantly smaller than that in control (*P*<0.01). Conversely, ZB significantly increased the cross-sectional area of quadriceps muscle fibers compared with model (*P*<0.05). There also was a leftward shift in the distribution of myofiber sizes in muscles from model mice compared with the nondiabetic control, and treatment with ZB improved the disadvantaged compared with model group (Fig. 4C).

### ZB increased serum IGF-1 and insulin, reduced serum lipid levels

**Figure 5:**
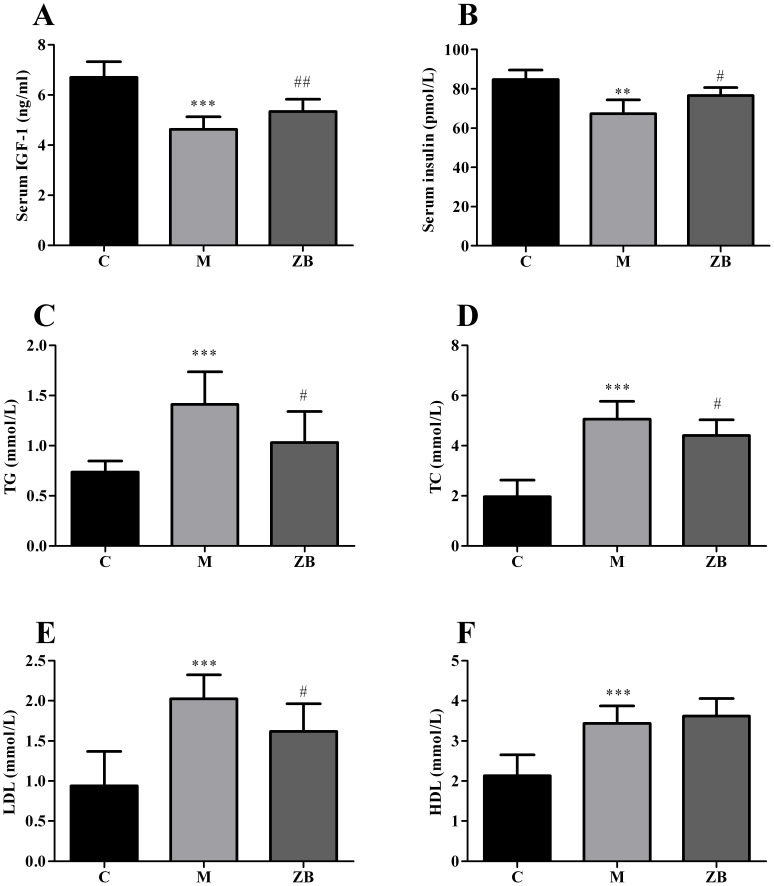
Effect of ZB on Serum IGF-1, insulin and Serum Lipid in diabetic mice. A: Serum IGF-1 levels of each group. B: Serum insulin levels of each group. C–F: The serum levels of triglyceride (TG), total cholesterol (TC), low-density lipoprotein cholesterol (LDL), high-density lipoprotein cholesterol (HDL). Data are expressed as the means±SD. ** *P*<0.01, *** *P*<0.001 vs. nondiabetic control. ^#^P<0.05, ^##^P<0.01 vs. Model group.

Serum insulin, IGF-1 were analysed with enzyme-linked immunosorbent assay (ELISA) kits and serum lipid levels were detected by semi-automatic biochemical analyzer. Treatment with ZB elevated the serum IGF-1 (*P*<0.01, Fig. 5A) and insulin (*P*<0.01, Fig. 5B) compared with model group. The serum levels of TC, TG, and LDL in the model mice were significantly greater than those of control group. However, treatment with ZB reduced the levels of TCs, TGs, and LDLs respectively (*P*<0.05, Fig. 5C, 5D, 5E), the results have significant differences. There was no statistical difference in the amounts of HDLs between the ZB group and model group (Fig. 5F).

### Effect of ZB on Akt/mTOR/FoxO signal pathways

**Figure 6:**
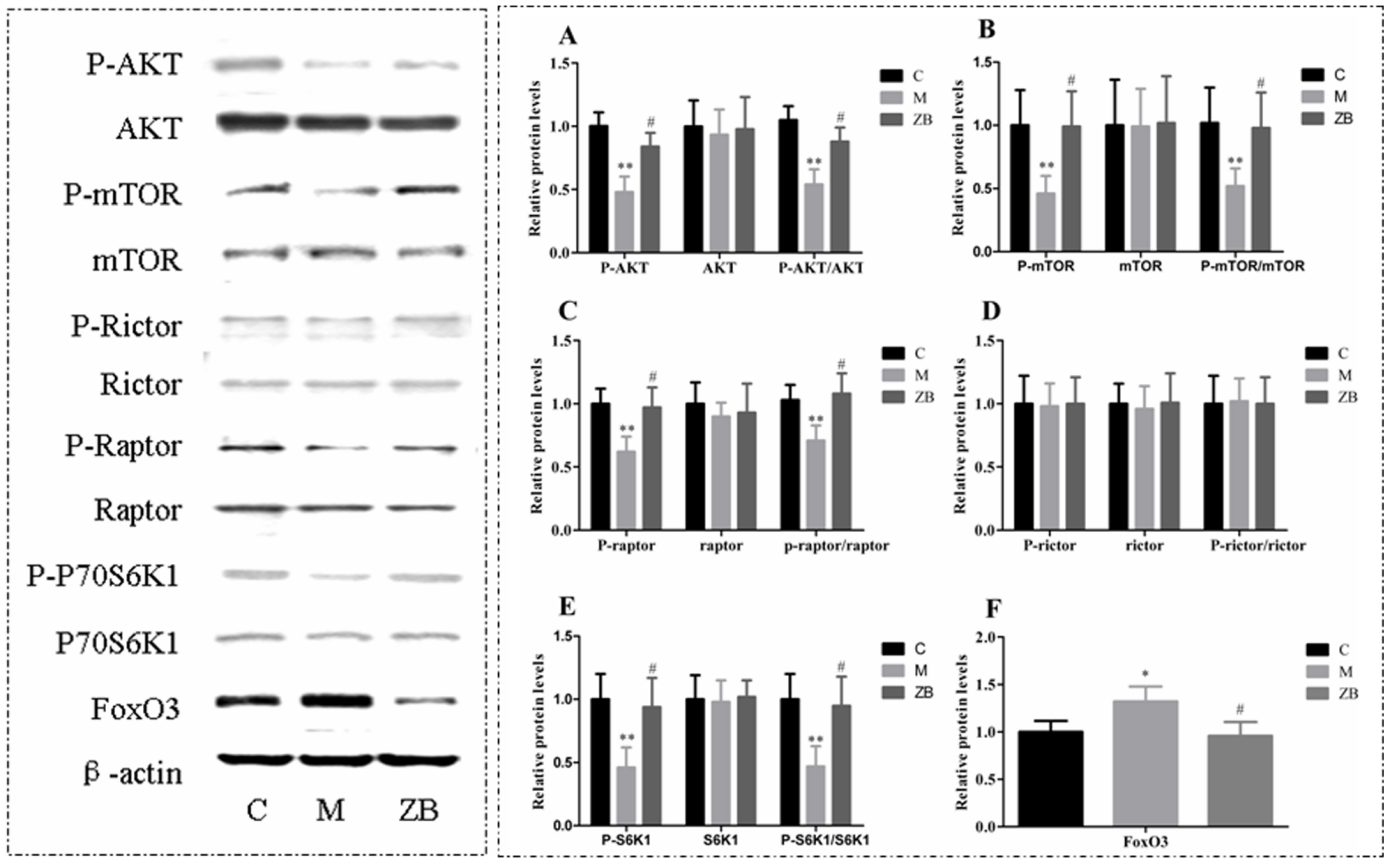
Effect of ZB on Akt/mTOR/FoxO3 signal pathways in diabetic mice. The relative protein expression levels of p-AKT/AKT (A), p-mTOR/mTOR (B), p-raptor/raptor (C), p-rictor/rictor (D), p-S6K1/S6K1 (E), FoxO3 (F) were evaluated by western blotting and normalized with β-actin. Data are expressed as the means±SD. * *P*<0.05, ** *P*<0.01 vs. nondiabetic control. ^#^ *P*<0.05 vs. Model group.

To investigate the molecular mechanism of the effect of ZB on reversal of muscle atrophy in diabetic mice, we explored the Akt/mTOR and FoxO3 signaling pathways which were closely related to the muscle protein synthesis and degradation by western blotting. The results showed that ZB could increase p-Akt (*P*<0.05, Fig. 6A), p-mTOR (*P*<0.05, Fig. 6B), p-Raptor (*P*<0.05, Fig. 6C), p-S6K1 (*P*<0.05, Fig. 6E) and reduced Foxo3 (*P*<0.05, Fig. 6F) compared with the model group. There was no statistical differences in p-rictor between ZB and model group (Fig. 6D). The results suggested that ZB could not only increase muscle protein synthesis by activating Akt/mTOR but also inhibit protein degradation by inactivation foxo3 protein.

## Discussion

In the present study, we generated a mouse model mimicking human type 2 diabetes with impaired insulin secretion and insulin resistance. The results showed that treatment with ZB considerably prevented body weight loss, muscle loss, muscle protein content reduction, and muscle fiber size decrease. Our results showed that ZB had a different effect on the gastrocnemius and quadriceps muscle. Since some researchers had reported that muscle fiber type distribution is not same in different organ, this might be explained by a different fiber type distribution. Moreover, ZB could increase grip strength and prolong the time on the rotarod compared with model group. In short, these findings indicated that administration of ZB could reverse muscle atrophy in the diabetic mice.

Skeletal muscle size depends upon a dynamic balance between synthesis and degradation of protein. And the two processes are tightly interrelated [10]. The present results showed that ZB could delay muscle atrophy in the diabetic mice. How did it work? To investigate the underline mechanisms of delaying diabetes muscle atrophy by ZB, we examined the effect of ZB on protein synthesis pathway by Western blotting. Since Akt/mTOR pathway is closely related with muscle hypertrophy and atrophy, the activation of the Akt/mTOR pathway can oppose muscle atrophy [13]. In addition, previous researches have demonstrated atrophic changes in skeletal muscle related to the reduced activity of Akt [48], [49]. Alternatively, increasing Akt expression has a potentially beneficial effect on diabetic skeletal muscles [4]. In this study, the result showed that ZB increased the level of phosphorylation Akt in the skeletal muscle. Akt can be activated by phosphorylation and subsequently activates downstream target of mTOR, which contains the key regulatory proteins involved in translation and protein synthesis [11], [16], [50]. Many researches have clearly confirmed that mTOR-mTORC1-S6K is an important pathway in regulating muscle protein synthesis [15], [18]–[20]. We demonstrated that ZB markedly increased the phosphorylations (p-) of mTOR, Raptor, S6K1 in the diabetic mice compared with the model. These findings suggested that ZB could promote skeletal muscle protein synthesis by activating Akt/mTOR pathway in diabetic mice.

In order to explore whether ZB could restrain degradation of protein, we evaluated the effect of ZB on FoxO3 transcription factor by Western blotting. The result showed that ZB reduced the expression of FoxO3 and increased p-Akt protein compared with the model. Previous study shows that FoxO3 transcription factor is essential for muscle atrophy [2]. Activation of FoxO3 leads to a dramatic loss of muscle mass. Alternatively, the muscle atrophy is prevented when FoxO3 is inactivated [22]. Generally FoxO3 induces expression of the atrophy-related ubiquitin ligases atrogin-1 and MuRF-1, which can reduce muscle mass [23], [24], [26]. Besides, FoxO3 is inactivated by Akt [25], [51]. Combining with the results we concluded that ZB could efficiently reduce degradation of skeletal muscle protein by regulating Akt/FoxO3 pathway in diabetic mice.

In the present study, we found out that treatment of diabetic mice with ZB effectively elevated serum IGF-1 levels compared with the model group. Previous studies have shown that IGF-1 is sufficient to induce skeletal muscle hypertrophy [7]. Besides, a study on transgenic mice shows that muscle-specific overexpression of an IGF-1 isoform locally expressed in skeletal muscle results in muscle hypertrophy [8]. Moreover, IGF-1 induces an increase in muscle mass by stimulating the phosphatidylinositol-3 kinase (PI3K)/Akt pathway [7], [9]. And Akt could induce the activation of mTOR, whose downstream targets, p70S6K and PHAS-1/4E-BP1, have been shown to promote protein synthesis [13], [50], [52]. Therefore, IGF-1 promotes protein synthesis mainly through the PI3K/Akt/mTOR pathways. On the other hand, IGF-1 can inhibit protein degradation by activation Akt. Akt represses the transcription factors of the FoxO family [52] and then block the upregulation of the ubiquitin-ligases MuRF1 and MAFbx, which are the key mediators of skeletal muscle atrophy [7], [11]. Therefore both the Akt/FoxO and Akt/mTOR pathways are regulated by IGF-1 [11]. Hence, stimulation of the IGF1/PI3K/Akt pathway would not only promote synthesis of protein but also dominantly suppress degradation of protein [10]. These findings insinuated that ZB could reverse muscle atrophy may be through increasing serum IGF-1 level in diabetic mice. The present study preliminary found that the level of IGF-1 in serum was upregulated by ZB extract, however, we failed to interpret the source of this hormone. Previously researches had showed that liver is a major IGF-1 producer, further study will be carried out to interpret the source of IGF-1.

The main reason that leads to muscle atrophy in type 2 diabetes is the reduction of insulin responsiveness in the muscle [53], [54]. Inhibition of mTOR leads to an impaired insulin action on glucose metabolism in skeletal muscle [55]. And insulin resistance causes muscle wasting by suppression of PI3K/Akt signaling pathway, which leads to activation of caspase-3 and the ubiquitin-proteasome proteolytic pathway, and resulted in muscle protein degradation [56]. Hence, improving insulin sensitivity can combat muscle wasting [57]. Our results showed that ZB could enhance glucose tolerance and elevate serum insulin levels in diabetic mice. Taken together, ZB could reverse muscle atrophy may be through ameliorating insulin sensitivity in diabetic mice.

Previous researches have confirmed that dyslipidaemia will lead to apoptosis and atrophy in skeletal muscle [58]. And we found out that serum levels of TCs, TGs, and LDLs in the model mice were significantly greater than those of control. However, treatment with ZB reduced the levels of TC, TG, and LDL. There is no significant difference in the amount of HDL between ZB and model group. Thus, ZB could reverse muscle atrophy may be through ameliorating dyslipidaemia in diabetic mice.

In summary, the present study demonstrated that the widely used traditional Chinese herb pair “ZB” was capable of reversing the muscle atrophy in diabetic mice, at least in part, through regulation of the Akt/mTOR/FoxO3 signal pathway. These findings indicated that ZB may act as a potential candidate drug in treatment of diabetic muscle atrophy.

## Supporting Information

Figure S1 **The total ion chromatography of ZB extracts (A) and nine standard substances (B) by RRLC-Q-TOF-MS in positive ESI mode.**
(DOC)
