# Supplementary material for: Reversal of Muscle Atrophy by Zhimu-Huangbai Herb-Pair via Akt/mTOR/FoxO3 Signal Pathway in Streptozotocin-Induced Diabetic Mice
Source: PLoS One. 2014 Jun 26;9(6):e100918. doi: 10.1371/journal.pone.0100918 (PMC4072704; doi:10.1371/journal.pone.0100918)
Supplement: Figure S1 — The total ion chromatography of ZB extracts (A) and nine standard substances (B) by RRLC-Q-TOF-MS in positive ESI mode. (DOC) [file pone.0100918.s001.doc]

**Supporting Information**

**
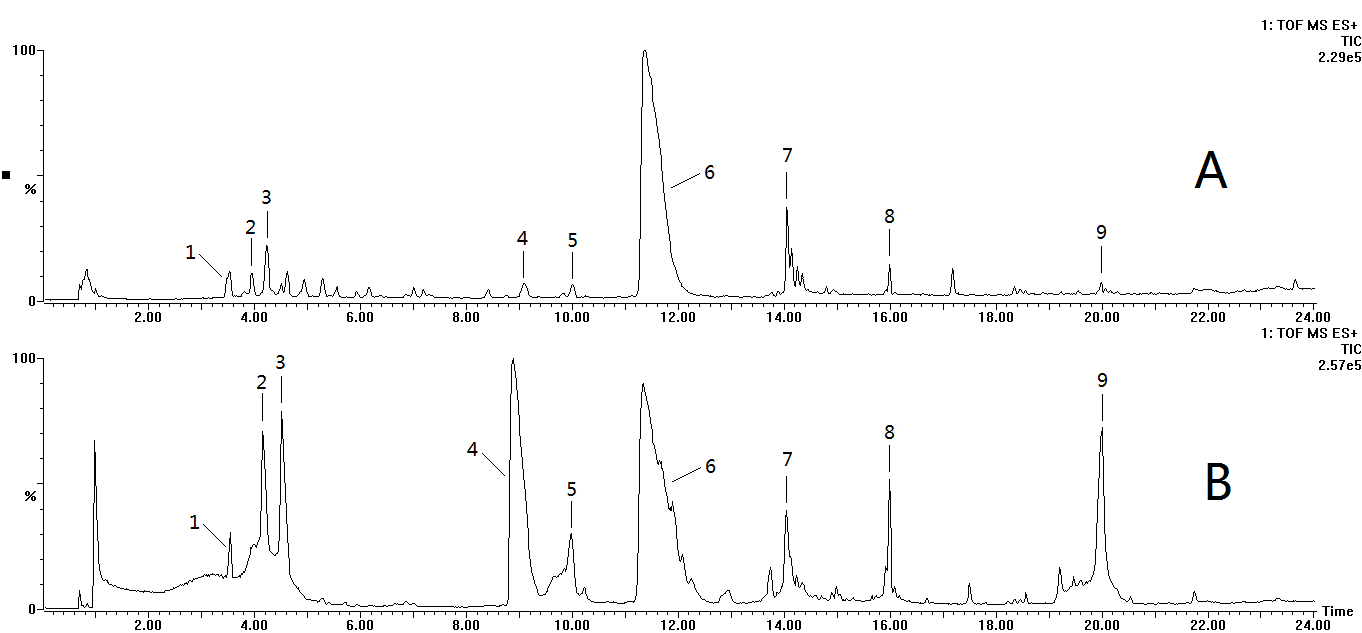
**

**Fig S1.** The total ion chromatography of ZB extracts (A) and nine standard substances (B) by RRLC-Q-TOF-MS in positive ESI mode.

The main components of the extracts were detected by UPLC/Q-TOF-MS system. According to retention time, molecular weight and the standard substances, nine of main compounds were identified as Fig S1: 1: Neomangiferin, 2: Phellodendrine, 3: Magnoflorine, 4: Jatrorrhizine, 5: Timosaponin E1, 6: Berberine, 7: Timosaponin B Ⅱ, 8: Timosaponin B, 9: Timosaponin A Ⅲ.
